# Is Hemoglobin Concentration a Linear Predictor of Mortality in Older Adults From Chinese Longevity Regions?

**DOI:** 10.3389/fpubh.2021.787935

**Published:** 2021-11-29

**Authors:** Jiaojiao Ren, Zhenghe Wang, Yujie Zhang, Peidong Zhang, Jianmeng Zhou, Wenfang Zhong, Xing Wang, Pingming Gao, Xiaoming Shi, Chen Mao

**Affiliations:** ^1^Affiliated Foshan Maternity & Child Healthcare Hospital, Southern Medical University, Foshan, China; ^2^Department of Epidemiology, School of Public Health, Southern Medical University, Guangzhou, China; ^3^The Second School of Clinical Medicine, Southern Medical University, Guangzhou, China; ^4^National Institute of Environmental Health, Chinese Center for Disease Control and Prevention, Beijing, China

**Keywords:** hemoglobin concentration, all-cause mortality, Cox models with restricted cubic spline curves, Chinese longevity regions, older adults

## Abstract

**Introduction:** The association patterns of hemoglobin (HB) concentrations with mortality among the longevity older adults are unclear. We aimed to evaluate the relationship among older adults form Chinese longevity regions.

**Methods:** We included 1,785 older adults aged ≥65 years (mean age, 86.7 years; 1,002 women, 783 men) from the community-based Chinese Longitudinal Healthy Longevity Survey. We estimated the hazard ratios (HRs) and 95% confidence intervals (CIs) for all-cause mortality using multivariable Cox proportional hazards models and Cox models with restricted cubic spline.

**Results:** In total, 999 deaths occurred during a median follow-up of 5.4 years from 2011 to 2017. Restricted cubic spline analysis found no non-linear association between HB concentrations and all-cause mortality after a full adjustment for covariates among the older adults form longevity regions (*p* > 0.05 for non-linearity). The risk for all-cause mortality was significantly higher in the groups with HB concentration of <11.0 g/dL (HR: 1.37, 95% CI: 1.10–1.70) and 11.0–12.0 g/dL (HR: 1.25, 95% CI: 1.01–1.54); the risk of all-cause mortality was significantly lower in the groups with HB concentration ≥14.0 g/dL (HR: 0.76, 95% CI: 0.60–0.97) compared with the reference group (13.0–13.9 g/dL).

**Conclusions:** Among older adults form Chinese longevity regions, HB concentrations were found to be inversely and linearly associated with all-cause mortality. Further prospective intervention trials are needed to confirm whether higher HB concentrations had a lower risk of mortality in these older adults.

## Introduction

An increase in life expectancy has emphasized anemia as a public health concern because of the associated healthcare needs and financial burden it incurs (1). Anemia is common among older adults with the estimated prevalence of 17% among individuals aged ≥65 years (2). A large cohort study has found that the prevalence of anemia increased with age from 4 to 6% in those aged 65–69 years to 13–14% in those aged ≥85 years (3). Anemia has been associated with a range of adverse events including falls, cognitive deficits, hospitalization, and mortality among older adults (4–7).

Anemia has been defined as hemoglobin (HB) concentrations of <12.0 g/dL and <13.0 g/dL in women and men, respectively, according to the World Health Organization criteria (8). Individual HB concentration is determined by environmental and genetic factors (9–11). Some studies have reported that relatively lower HB concentrations were predictors of increased risk of mortality, which were due to decreased oxygen carrying capacity causing left ventricular hypertrophy and ischemia (12, 13). More recently, several prospective cohort studies have indicated that a non-linear association exists between HB concentrations and all-cause mortality. For example, there were U- or J-shaped associations between HB concentration and all-cause mortality in the studies of women aged 20–39 years, women aged >40 years, and postmenopausal women (14–16).

Anemia may be prevalent in the general population, particularly in older adults (17). The effect of HB concentrations is associated with infection, autoimmune disease, and chronic kidney disease (18). However, establishing whether HB concentration is an independent risk factor for mortality among older adults needs to be further explored. The previous literature studies have aimed at specific populations (hemodialysis patients or young women), and studies of community-based older adults are rare. In addition, most studies identify HB concentrations as qualitative variables to assess HB concentrations with mortality, studies defined HB concentrations as a continuous variable using Cox models with restricted cubic spline, which is an essential method for exploring linear or non-linear associations, are limited; most studies have paid close attention the association of lower HB concentrations with mortality, but the effect of higher HB concentrations on mortality among older adults is still unclear.

In the current study, we aimed to evaluate the relationship between HB concentrations and all-cause mortality among older adults aged ≥65 years form Chinese longevity regions, using community-based cohort data from the Chinese Longitudinal Healthy Longevity Survey (CLHLS).

## Materials and Methods

### Study Setting and Participants

**Figure 1:**
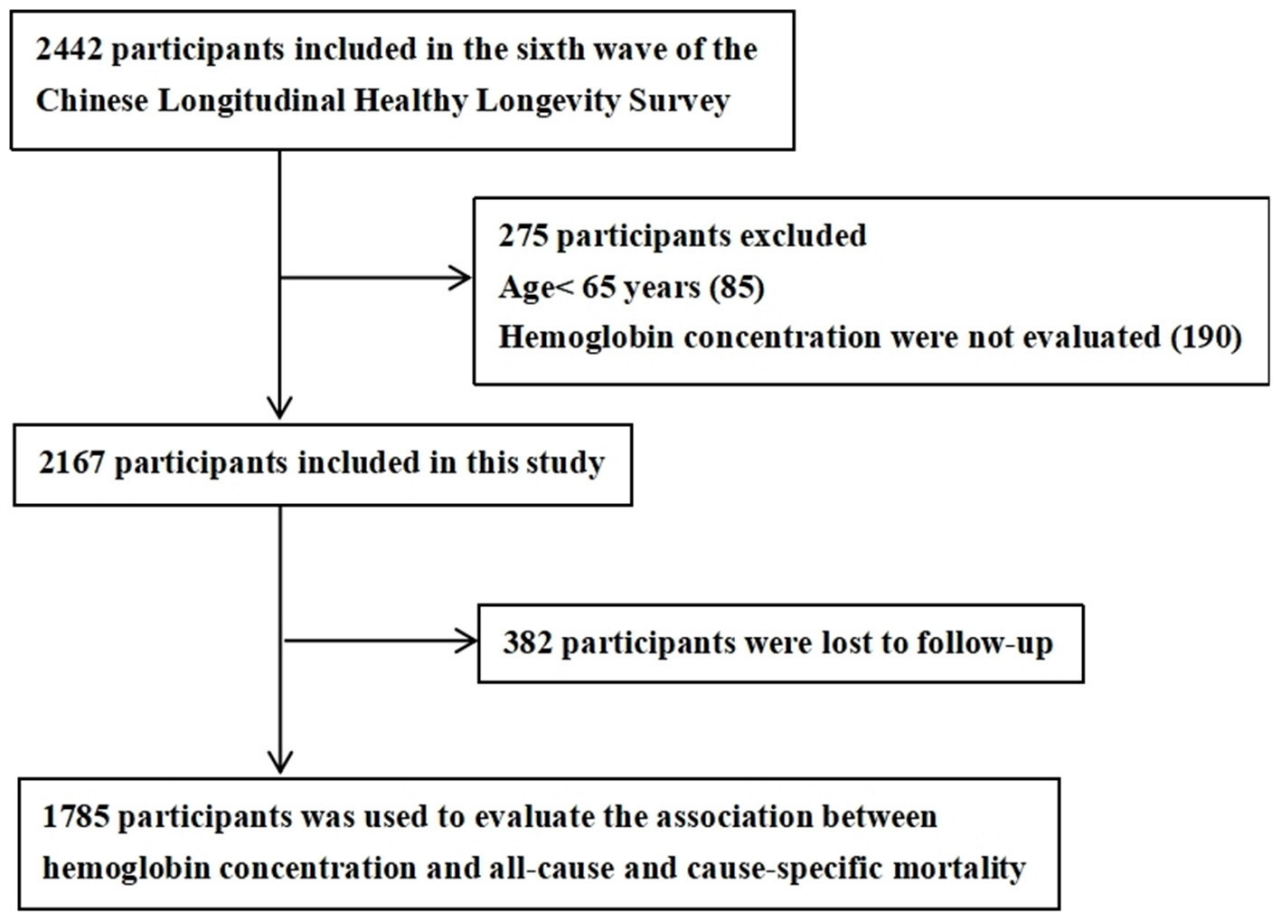
A flowchart of the participant enrollment in the study.

The data used in this study were extracted from the CLHLS, a prospective, community-based cohort study with a median 5.4-year follow-up period (2011–2017), details of which are available elsewhere (19, 20). In brief, participants were enrolled from the sixth wave (2011) of CLHLS assessments that are performed in eight Chinese regions (including Yong Fu county, Ma Yang county, Chen Mai county, Rudong county, Xia Yi county, Zhong Xiang city, Shanshui city, and Lai Zhou city). The standards of Chinese longevity regions as follows: the existing centenarians in the region account for more than 7.0/100,000 of the total population; the average life expectancy of the regional population is 3 years higher than the national level, and the proportion of the older adults aged ≥80 years accounts for more than 1.4% of the total population. We obtained the baseline health examination and blood test data of all adults aged ≥65 years surveyed within the study period. After excluding cases with missing information on key variables, the final sample consisted of 1,785 older adults (Figure 1). Participant information was systematically collected during face-to-face interviews conducted by trained staff. The study was approved by the Research Ethics Committee of Peking University (IRB00001052-13074) and informed consent of all participants has been obtained.

### Laboratory Methods

Medical professionals collected blood samples from all eligible participants. Data on the blood-related variables of interest were obtained from the baseline survey (2011–2012). The blood samples were transported and stored at −80°C in Beijing. HB, total cholesterol (TC), C-reactive protein (CRP) levels, and serum creatinine (Scr) levels were measured using the HB cyanide test, enzymatic colorimetric test, immunoturbidimetric assay, and enzymatic method, respectively (21).

### Ascertainment of HB Concentrations

To assess whether the risk of mortality changed at different HB concentrations in older adults form longevity regions, we categorized HB concentrations into five categories (<11.0 g/dL, 11.0–11.9 g/dL, 12.0–12.9 g/dL, 13.0–13.9 g/dL, and ≥14.0 g/dL). An HB concentration of 13.0–13.9 g/dL was selected as the reference group for comparison based on previous studies (12).

### Ascertainment of Covariates

Data on variables that may confound the relationship between HB concentrations and all-cause mortality were extracted from answers to the questionnaire and biochemistry tests (22). The covariates of interest were demographic characteristics (age, sex, residence, education level, marital status, and economic status); lifestyle factors (smoking status, drinking status, tea drinking status, food diversity score, and regular exercise uptake); disease and psychological conditions; history of chronic diseases (hypertension, diabetes, stroke, heart disease), depressive symptoms score; biochemical test findings [body mass index (BMI), white blood cell (WBC) count, the mean corpuscular volume (MCV) estimates, the levels of CRP and TC, platelet count, and eGFR]; findings from the assessment of the activities of daily living (ADL); and the Mini-Mental State Examination (MMSE) score. To reduce the potential impact of inferential bias, we used multiple imputation methods to correct for missing values of the relevant covariates (23).

### Ascertainment of Deaths

The survival outcomes of the participant were confirmed in the seventh (2014) and eighth (2017) waves of the CLHLS. The date of death was confirmed by the participants' next-of-kin and local physician. We calculated the survival time from the interview date at baseline in 2011 to the date of death. “Lost to follow-up” status applied to individuals who could not be found or contacted. The data for the participants who were alive until the 2017 survey were censored at that date.

### Statistical Analysis

The variables were summarized to describe categorical variables as number (percentage), normally distributed variables as mean [standard deviation (SD)], and skew distribution variable as median (interquartile range). The Cox proportional hazards model was presented to evaluate hazard ratios (HRs) and 95% CI, as measures of the association of HB concentrations with all-cause mortality, adjusted for potential confounding factors. Model 1 was not adjusted for any covariates. Model 2 included adjustments for age (years), sex (male or female), residence (urban or rural), education level (years), marital status (married or unmarried), and economic status (rich, general, or poor). Model 3 included additional adjustments for smoking status (current smoker or non-smoker); drinking status (current drinker or non-drinker); tea drinking status (yes or no); food diversity score (cereals, vegetables, fruits, meat, fish, eggs, milk, beans, and grease, 1 point for each type of food consumed for each individual in 1 week); regular exercise uptake (yes or no); restricted ADL (bathing, getting out of bed, dressing, walking across a room, eating, and continence status, any of six activities not independently completed defined as restricted ADL); self-reported diagnosis of hypertension (yes or no), diabetes (yes or no), stroke (yes or no), or heart disease (yes or no); MMSE score (the measure of cognitive impairment, no cognitive impairment [25 ≤ MMSE score ≤ 30]); depressive symptom score (a five item scale, and a score from 0 to 4 assigned to each response) (24); Model 4, the final model, included additional adjustments for BMI (kg/m^2^); CRP levels (mg/L); eGFR (ml/min/1.73 m^2^; calculated based on Scr according to Chronic Kidney Disease Epidemiology Collaboration); WBC (10^9^/l); MCV estimates (fl); platelet count (10^9^/l), and TC levels (mmol/L).

Furthermore, we performed restricted cubic spline curves based on multivariable Cox proportional hazards models using HB concentration as a continuous variable to examine linear or non-linear associations between HB concentrations and all-cause mortality in the fully adjusted model. We conducted stratified analyses to assess potential modification effects of the following factors: age, sex, education level, economic status, smoking, drinking, and tea drinking status, regular exercise uptake, and BMI. Anticipating the complex association between HB concentrations and all-cause mortality, we conducted three types of sensitivity analyses. First, we excluded the participants that died in the first year of the study to minimize the potential effect of reverse causation. Second, we excluded participants with a history of hypertension, diabetes, stroke, and heart disease to reduce spurious increases in the risk of mortality. Third, we excluded participants who had low BMI (<18.5 kg/m^2^) to eliminate the impact of malnutrition. A two-tailed *p*-value of <0.05 was considered indicative of a statistically significant finding. Statistical analysis was performed using R software version 3.5.0 (R Center for Statistical Computing, Vienna, Austria).

## Results

### Baseline Characteristics

Of 1,785 participants form longevity regions, the mean age was 86.7 years (*SD*: 12.2), and 1,002 (56.1%) were women. Overall, the mean HB concentration was 12.2 g/dL (*SD*: 23.1). With the increasing HB concentrations, the proportion of female participants, restricted ADL, and mean age decreased. Conversely, participants with higher HB concentrations were more likely to be current smokers or drinkers; perform regular exercise; reside in rural environments; have better economic status; and have higher levels of eGFR, MCV, and platelet count (Table 1).

**Table 1:** Baseline characteristics of respondents according to HB concentrations.

**Characteristics**	**HB concentrations, g/dL**					**Total**
	**<11.0**	**11.0–11.9**	**12.0–12.9**	**13.0–13.9**	**≥14.0**	
	**(*n* = 441)**	**(*n* = 360)**	**(*n* = 322)**	**(*n* = 297)**	**(*n* = 365)**	
Demographic characteristics					
Age, mean (SD), y	92.1 (10.6)	89.3 (11.2)	86.2 (12.1)	84.5 (11.9)	79.6 (11.6)	86.7 (12.2)
Women, *n* (%)	324 (73.5)	245 (68.1)	199 (61.8)	138 (46.5)	96 (26.3)	1,002 (56.1)
Education time, mean (SD), y	0.9 (2.1)	1.3 (2.9)	1.9 (2.9)	2.4 (3.4)	2.9 (3.3)	1.8 (3.0)
Married, *n* (%)	347 (78.7)	248 (68.9)	207 (64.3)	165 (55.6)	152 (41.6)	1,119 (62.7)
Residence					
Urban	90 (20.4)	70 (19.4)	52 (16.1)	41 (13.8)	41 (11.2)	294 (16.5)
Rural	351 (79.6)	290 (80.6)	270 (83.9)	256 (86.2)	324 (88.8)	1,491 (83.5)
Economic status					
Rich	48 (10.9)	59 (16.4)	65 (20.2)	67 (22.6)	68 (18.6)	307 (17.2)
General	343 (77.8)	260 (72.2)	215 (66.8)	202 (68.0)	256 (70.1)	1,276 (71.5)
Poor	50 (11.3)	41 (11.4)	42 (13.0)	28 (9.4)	41 (11.2)	202 (11.3)
Lifestyle factors					
Current smoker, *n* (%)	33 (7.5)	39 (10.8)	43 (13.4)	65 (21.9)	92 (25.2)	272 (15.2)
Current drinker, *n* (%)	22 (5.0)	45 (12.5)	44 (13.7)	60 (20.2)	96 (26.3)	267 (15.0)
Tea drinking, *n* (%)	111 (25.2)	145 (40.3)	132 (41.0)	124 (41.8)	145 (39.7)	657 (36.8)
Food diversity score, mean (SD)	7.3 (1.3)	7.4 (1.3)	7.3 (1.3)	7.3 (1.3)	7.2 (1.2)	7.3 (1.3)
Regular exercise, *n* (%)	37 (8.4)	40 (11.1)	45 (14.0)	40 (13.5)	58 (15.9)	220 (12.3)
Disease and psychological conditions					
Restricted ADL, *n* (%)	123 (27.9)	84 (23.3)	72 (22.4)	47 (15.8)	51 (14.0)	377 (21.1)
MMSE score, mean (SD)	20.1 (10.1)	22.1 (9.4)	22.6 (9.1)	23.0 (8.8)	24.8 (8.1)	22.4 (9.3)
Depressive score, mean (SD)	11.1 (2.1)	11.1 (2.1)	11.1 (2.0)	11.2 (1.7)	11.2 (1.9)	11.1 (2.0)
Hypertension, *n* (%)	85 (19.3)	106 (29.4)	103 (32.0)	85 (28.6)	66 (18.1)	445 (24.9)
Diabetes, *n* (%)	7 (1.6)	10 (2.8)	5 (1.6)	6 (2.0)	5 (1.4)	33 (1.8)
Stroke, *n* (%)	27 (6.1)	30 (8.3)	26 (8.1)	26 (8.8)	41 (11.2)	150 (8.4)
Heart disease, *n* (%)	31 (7.0)	30 (8.3)	29 (9.0)	30 (10.1)	20 (5.5)	140 (7.8)
Biochemical indicators					
BMI, mean (SD), kg/m^2^	19.7 (5.8)	20.4 (5.1)	23.4 (7.8)	22.1 (4.4)	23.6 (8.1)	21.7 (13.2)
CRP, median (IQR), mg/L	1.0 (0.0–4.0)	1.0 (0.0–3.0)	1.0 (0.0–2.0)	1.0 (0.0–2.0)	1.0 (0.0–2.0)	1.0 (0.0–3.0)
eGFR, mean (SD), ml/min/1.73 m^2^	62.7 (20.6)	71.1 (18.5)	76.9 (16.4)	80.0 (17.0)	84.2 (16.1)	74.3 (19.6)
WBC count, mean (SD), 10^9^/l	5.7 (2.1)	5.7 (1.8)	5.5 (2.0)	5.5 (1.7)	5.4 (1.4)	5.6 (1.8)
MCV, mean (SD), fl	92.4 (10.2)	94.4 (7.2)	94.5 (6.7)	96.6 (6.2)	99.3 (17.3)	95.3 (10.9)
Platelet count, mean (SD), 10^9^/l	190.1 (91.8)	201.1 (134.8)	207.5 (95.6)	214.1 (93.9)	234.8 (91.2)	208.6 (103.8)
TC, mean (SD), mmol/L	4.0 (1.0)	4.3 (1.0)	4.5 (1.0)	4.4 (1.0)	4.4 (0.9)	4.3 (1.0)

*SD, standard deviation; IQR, interquartile range; HB, hemoglobin; ADL, activities of daily living; MMSE, Mini-Mental State Examination; BMI, body mass index; CRP, C-reactive protein; eGFR, Estimated Glomerular Filtration Rate; WBC, white blood cell; MCV, mean corpuscular volume; TC, total cholesterol*.

### HB Concentrations and All-Cause Mortality

During a median of 5.4 years' follow-up (interquartile range: 4.4–6.1 years), we identified 999 deaths (men: 623, women: 376), accounting for 56.0% of all participants. Table 2 presents data of the association of stratified HB concentrations with all-cause mortality in older adults form longevity regions. Compared with the reference group (13.0–13.9 g/dL), groups with the lowest (<11.0 g/dL) and highest (≥14.0 g/dL) HB concentrations had fully adjusted HRs of 1.37 (95% CI: 1.10–1.70) and 0.76 (95% CI: 0.60–0.97), respectively, for all-cause mortality.

**Table 2:** HRs (95% CI) for all-cause mortality according to HB concentrations in the older adults form Chinese longevity regions.

**HB concentrations, g/dL**	**Deaths/*n***	**Model 1^a^**		**Model 2^b^**		**Model 3^c^**		**Model 4^d^**	
		**HR (95% CI)**	***P***	**HR (95% CI)**	***P***	**HR (95% CI)**	***P***	**HR (95% CI)**	***P***
<11.0	312/441	1.92 (1.58–2.33)	<0.001	1.37 (1.12–1.67)	0.002	1.52 (1.24–1.86)	<0.001	1.37 (1.10–1.70)	0.005
11.0–11.9	228/360	1.50 (1.22–1.84)	<0.001	1.22 (1.00–1.51)	0.057	1.32 (1.07–1.62)	0.011	1.25 (1.01–1.54)	0.043
12.0–12.9	173/322	1.08 (0.87–1.35)	0.473	0.99 (0.80–1.24)	0.956	0.98 (0.79–1.22)	0.856	0.98 (0.79–1.22)	0.856
13.0–13.9	150/297	1.00 (reference)	–	1.00 (reference)	–	1.00 (reference)	–	1.00 (reference)	–
≥14.0	136/365	0.66 (0.53–0.84)	0.001	0.82 (0.65–1.04)	0.100	0.76 (0.60–0.96)	0.019	0.76 (0.60–0.97)	0.025

a *No adjustment*.

b *Adjusted for age, sex, education time, marital status, residence, and economic status*.

c *Additionally adjusted for smoking status, drinking status, tea drinking, food diversity score, regular exercise, restricted ADL, self-reported diagnosed hypertension, diabetes, stroke, heart disease, MMSE score, and depressive symptom score*.

d *Additionally adjusted for BMI, CRP, eGFR, WBC count, MCV, platelet count, and TC*.

*HB, hemoglobin; ADL, activities of daily living; MMSE, Mini-Mental State Examination; BMI, body mass index; CRP, C-reactive protein; eGFR, Estimated Glomerular Filtration Rate; WBC, white blood cell; MCV, mean corpuscular volume; TC, total cholesterol*.

**Figure 2:**
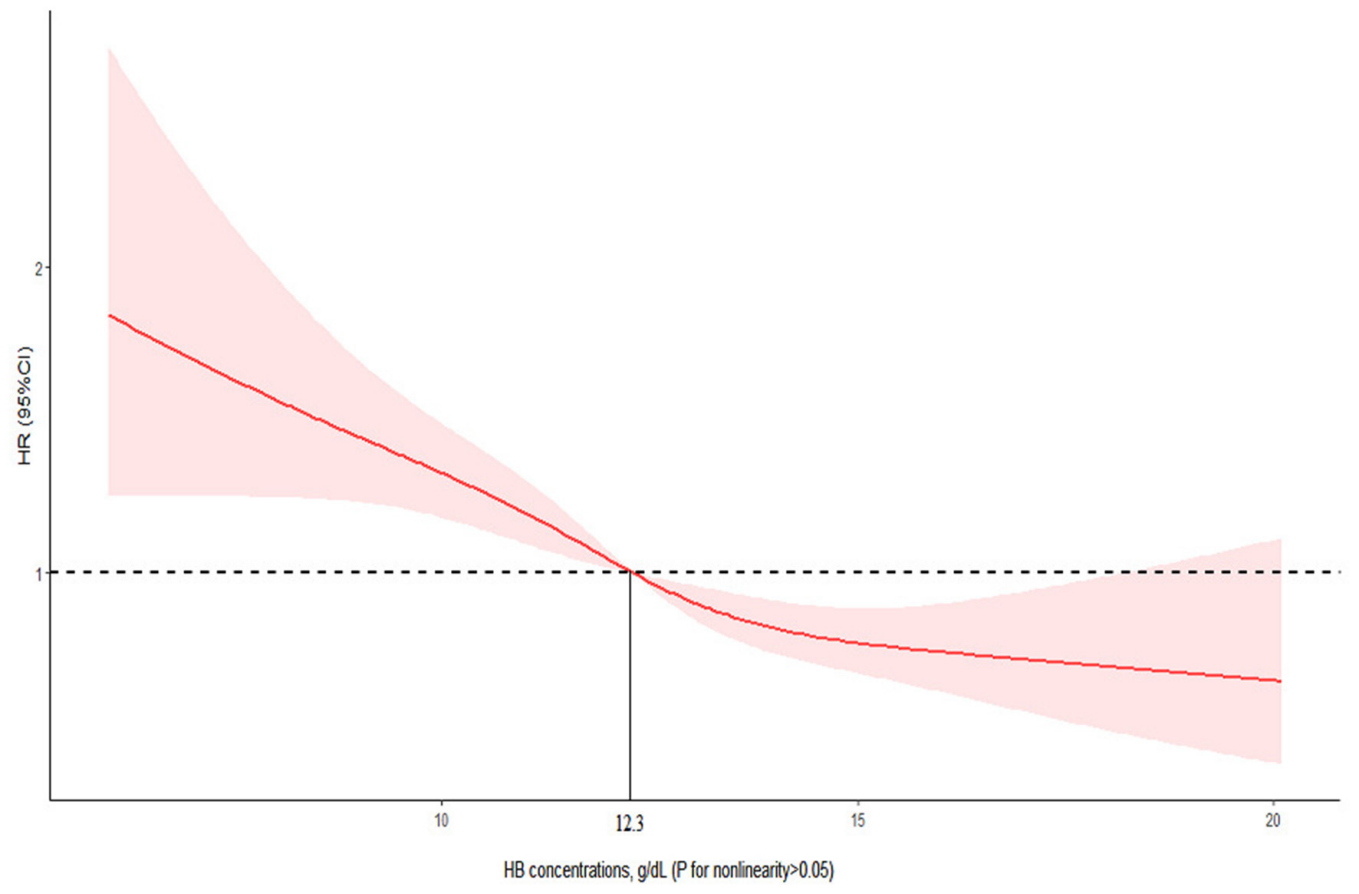
Association of HB with all-cause mortality among the older adults form Chinese longevity regions. Cox models with restricted cubic spline curves adjusted for age, sex, education time, marital status, residence, economic status, smoking status, drinking status, tea drinking, food diversity score, regular exercise, ADL, self-reported diagnosed hypertension, diabetes, stroke, heart disease, MMSE score, depressive symptom score, BMI, CRP, eGFR, WBC count, MCV, platelet count, and TC.

Similar to the results above, the restricted cubic spline curves analysis revealed no non-linear association of HB concentrations with all-cause mortality (*p* = 0.351 for non-linearity; Figure 2). Participants with HB concentrations lower than 12.3 g/dL had a significantly increased risk of all-cause mortality and those with HB concentrations of higher than 12.3 g/dL had a significantly lower risk of all-cause mortality. However, the HB concentration of 12.0–12.9 g/dL was not associated with all-cause mortality.

**Figure 3:**
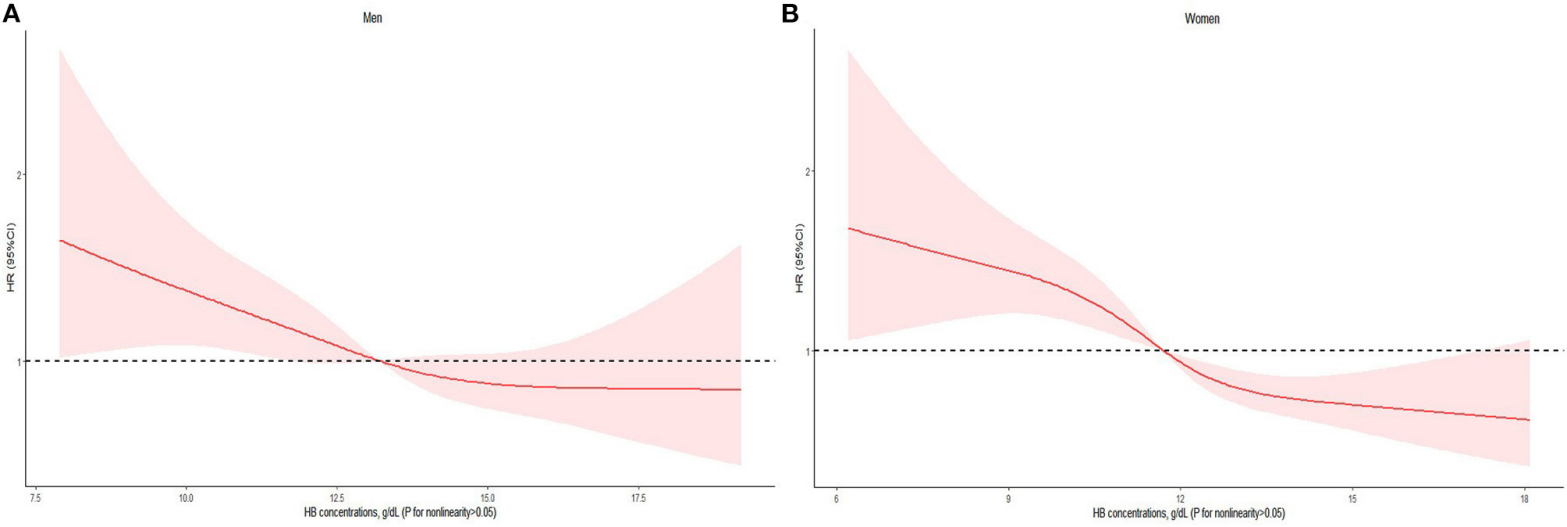
HB on a continuous scale and risk of all-cause mortality in the older adults form Chinese longevity regions stratified by sex: **(A)** men, **(B)** women. Cox models with restricted cubic spline curves adjusted for age, education time, marital status, residence, economic status, smoking status, drinking status, tea drinking, food diversity score, regular exercise, ADL, self-reported diagnosed hypertension, diabetes, stroke, heart disease, MMSE score, depressive symptom score, BMI, CRP, eGFR, WBC count, MCV, platelet count, and TC.

We further analyzed the association between HB concentrations and all-cause mortality by sex owing to the different HB reference criteria for men and women. A similar linear association was also observed between HB concentrations and all-cause mortality for both men and women (Figure 3). For men, the risk of all-cause mortality was significantly lower with the highest HB concentration of ≥14.0 g/dL in the fully adjusted model (HR: 0.69, 95% CI: 0.50–0.96); and for women, the risk of all-cause mortality was significantly higher with the lowest HB concentrations of <11.0 g/dL in the fully adjusted model (HR: 1.42, 95% CI: 1.11–1.82) (Table 3). Furthermore, there was no significant interaction effect between HB concentrations and all-cause mortality by age, education time, economic status, smoking status, drinking status, tea drinking status, regular exercise uptake, and BMI (Table 4). The sensitivity analyses revealed that the association between HB concentrations and all-cause mortality was largely similar to the primary results when excluding individuals who died in the first year; those with a history of hypertension, diabetes, stroke, and heart disease; and those who had low BMI (<18.5 kg/m^2^) based on the fully adjusted model (Table 5).

**Table 3:** HRs (95% CI) for all-cause mortality according to HB concentrations among the older adults form Chinese longevity regions by sex.

**HB concentrations, g/dL**	**Deaths/*n***	**Model 1^a^**		**Model 2^b^**		**Model 3^c^**		**Model 4^d^**	
		**HR (95% CI)**	***P***	**HR (95% CI)**	***P***	**HR (95% CI)**	***P***	**HR (95% CI)**	***P***
**Men**								
<11.0	91/117	2.43 (1.79–3.31)	<0.001	1.37 (0.99–1.89)	0.055	1.31 (0.94–1.83)	0.106	1.09 (0.77–1.56)	0.632
11.0–11.9	70/115	1.57 (1.13–2.18)	0.007	1.20 (0.86–1.67)	0.281	1.22 (0.87–1.70)	0.253	1.18 (0.84–1.66)	0.338
12.0–12.9	58/122	1.01 (0.72–1.43)	0.943	0.85 (0.60–1.20)	0.352	0.74 (0.52–1.06)	0.099	0.75 (0.53–1.08)	0.120
13.0–13.9	75/160	1.00 (reference)	–	1.00 (reference)	–	1.00 (reference)	–	1.00 (reference)	–
≥14.0	82/269	0.57 (0.42–0.78)	<0.001	0.73 (0.53–1.00)	0.053	0.64 (0.46–0.89)	0.007	0.69 (0.50–0.96)	0.026
**Women**								
<11.0	221/324	1.54 (1.23–1.93)	<0.001	1.28 (1.02–1.61)	0.033	1.46 (1.16–1.85)	0.001	1.42 (1.11–1.82)	0.005
11.0–11.9	158/245	1.30 (1.02–1.65)	0.036	1.17 (0.92–1.49)	0.208	1.27 (0.99–1.63)	0.053	1.28 (0.99–1.64)	0.053
12.0–12.9	114/199	1.00 (reference)	–	1.00 (reference)	–	1.00 (reference)	–	1.00 (reference)	–
13.0–13.9	76/138	0.94 (0.70–1.26)	0.669	0.89 (0.66–1.19)	0.429	0.87 (0.64–1.17)	0.336	0.85 (0.63–1.15)	0.292
≥14.0	54/96	0.95 (0.69–1.31)	0.749	0.90 (0.65–1.25)	0.537	0.81 (0.58–1.13)	0.210	0.76 (0.55–1.07)	0.115

a *No adjustment*.

b *Adjusted for age, education time, marital status, residence, and economic status*.

c *Additionally adjusted for smoking status, drinking status, tea drinking, food diversity score, regular exercise, restricted ADL, self-reported diagnosed hypertension, diabetes, stroke, heart disease, MMSE score, and depressive symptom score*.

d *Additionally adjusted for BMI, CRP, eGFR, WBC count, MCV, platelet count, and TC*.

*HB, hemoglobin; ADL, activities of daily living; MMSE, Mini-Mental State Examination; BMI, body mass index; CRP, C-reactive protein; eGFR, Estimated Glomerular Filtration Rate; WBC, white blood cell; MCV, mean corpuscular volume; TC, total cholesterol*.

**Table 4:** Subgroup analyses of the association between HB concentrations and all-cause mortality risk in the older adults form Chinese longevity regions.

**Subgroup**	**Deaths/No. of participants**	**HR (95% CI)**	***P*-interaction**
Age, y			0.193
65–74	67/382	0.99 (0.98–1.01)	
75–84	145/412	1.00 (0.99–1.01)	
85–94	280/414	1.00 (0.99–1.01)	
≥95	507/577	0.99 (0.98–0.99)	
Sex			0.597
Men	376/783	0.99 (0.99–1.00)	
Women	623/1,002	0.99 (0.98–0.99)	
Education time, y			0.212
0	751/1,160	0.99 (0.99–0.99)	
≥1	248/625	0.99 (0.99–1.00)	
Economic status			0.273
Rich	154/307	1.00 (0.99–1.01)	
General	721/1,276	0.99 (0.98–0.99)	
Poor	124/202	0.99 (0.98–1.00)	
Current smoker			0.775
Yes	109/272	0.99 (0.99–0.99)	
No	890/1,513	0.99 (0.98–1.00)	
Current drinker			0.508
Yes	121/267	1.00 (0.98–1.01)	
No	878/1,518	0.99 (0.99–1.00)	
Tea drinking			0.258
Yes	337/657	1.00 (0.99–1.00)	
No	662/1,128	0.99 (0.99–0.99)	
Regular exercise			0.424
Yes	89/220	0.99 (0.98–1.01)	
No	910/1,565	0.99 (0.99–1.00)	
BMI, kg/m^2^			0.631
<18.5	310/435	0.99 (0.98–0.99)	
18.5–23.9	524/936	0.99 (0.99–1.00)	
≥24.0	165/414	0.99 (0.99–1.00)	

*HR, hazard ratio; CI, confidence interval; BMI, body mass index*.

**Table 5:** Sensitivity analyses for the relationship between HB concentrations and all-cause mortality risk in the older adults form Chinese longevity regions.

**HB Concentrations, g/dL**	**HR (95% CI)**					
	**Deaths/*n***	**Excluding the participants who died in the first year**	**Deaths/*n***	**Excluding the participants with a history of hypertension, diabetes, stroke, and heart disease**	**Deaths/*n***	**Excluding the participants who had low BMI (<18.5 kg/m^**2**^)**
<11.0	224/353	1.42 (1.14–1.77)^*^	212/318	1.30 (0.99–1.70)	182/272	1.20 (0.93–1.56)
11.0–11.9	175/307	1.25 (0.99–1.57)	133/217	1.31 (0.99–1.72)	146/244	1.13 (0.88–1.46)
12.0–12.9	146/295	1.01 (0.80–1.28)	92/184	0.92 (0.69–1.24)	123/248	0.92 (0.72–1.19)
13.0–13.9	129/276	1.00 (reference)	94/176	1.00 (reference)	122/249	1.00 (reference)
≥14.0	123/352	0.75 (0.58–0.96)^*^	87/251	0.69 (0.51–0.93)^*^	112/328	0.67 (0.51–0.87)^*^

* *P ≤ 0.05*.

*HR, hazard ratio; CI, confidence interval*.

*Sensitivity analyses were based on the fully adjusted model*.

## Discussion

In the present study of community-dwelling older adults form longevity regions, we found the presence of independent associations of HB concentrations with all-cause mortality. The participants with the highest HB concentration (≥14.0 g/dL) had a lowest risk of all-cause mortality, and those with lower HB concentration (<12.0 g/dL) had a higher risk of all-cause mortality. We observed inversely linear association of HB concentrations with all-cause mortality using Cox models with restricted cubic spline curves.

Early studies have consistently elaborated the association between anemia and all-cause mortality in the general population, and the HB concentrations according to the WHO criteria in these studies were also generally categorized by anemia and non-anemia. For instance, a study by Denny reported the HR of 8-year mortality as 1.70 (95% CI: 1.5–2.0) among participants with anemia (25); a separate study has shown a similar increase in the all-cause mortality risk associated with anemia (HR: 1.39, 95% CI: 1.15–1.69) (26). However, these might not clarify an association of higher HB concentration (≥14.0 g/dL) with mortality.

Recent prospective cohort studies provided evidence of an U-shaped association between HB concentrations and all-cause mortality among women (27). Our community-based cohort study indicated an inconsistent linear association of HB concentration with all-cause mortality among older adults form longevity regions. We found that lower HB concentrations (<12.0 g/dL) were associated with increased all-cause mortality risk. A possible mechanism through which lower HB concentrations increased the risk of all-cause mortality in older adults could be that it led to reduced oxygen delivery and inflammatory conditions (28, 29). However, highest HB concentrations (≥14.0 g/dL) could decrease the risk of all-cause mortality. One possible explanation could be that the causes of the effects of HB concentrations among the older adults are complex, especially for longevity regions (30). Our study included 1,223 older adults aged ≥80 years (68.5%) and the adaptive changes made by their bodies during the aging process may not increase the mortality risk with higher HB concentrations. Alternatively, the inter-study variations in the definition of HB concentration reference cutoffs could explain the association (31). Moreover, the ethnicity of older adults differed between cohorts.

There are still some concerns when assessing the association between HB concentrations and mortality. The most important concern is the reverse causality related to mortality. For instance, chronic diseases can lead to a higher mortality rate for older adults, and are related to HB concentrations, which may falsely increase the estimated risk of mortality (32). Therefore, we excluded the participants who died in the first year to minimize the bias of reverse causality and found similar results. Another concern is that the possible residual confounding may confuse the association between HB concentrations and mortality. For example, sex is an extremely important factor associated with the reference cutoff values of HB concentrations (7). However, when the analyses were stratified by sex, the linear association did not change. Additionally, we adjusted for potential confounders, including demographic characteristics, lifestyle factors, disease and psychological conditions, and biochemical indicators, and our results also suggested a linear relationship between HB concentrations and mortality among older adults.

The strengths of this study include the use of a large community-based population, the detailed information on potential factors, and comprehensive analyses to examine the relationship of HB concentrations with all-cause mortality among older adults form longevity regions. In addition, we revealed the linear associations of HB concentrations with mortality using Cox models with restricted cubic spline curves. Nevertheless, our study also has some limitations. First, this is an observational research design and the causal relationship between HB concentrations and mortality cannot be determined. Second, HB concentration was measured only at baseline, so the study could not investigate the influence of HB concentration changes on the mortality risk during the follow-up period. Third, the relatively small sample size may have resulted in the low statistical power of some subgroup analyses.

## Conclusion

This study showed that the association of HB concentrations with all-cause mortality was inversely linear in older adults form longevity regions. These findings need prospective intervention trials to confirm that higher HB concentrations can decrease mortality risk in older adults form longevity regions.

## Data Availability Statement

The raw data supporting the conclusions of this article will be made available by the authors, without undue reservation.

## Ethics Statement

The studies involving human participants were reviewed and approved by the Research Ethics Committee of Peking University (IRB00001052-13074). The patients/participants provided their written informed consent to participate in this study.

## Author Contributions

CM and XS contributed to conception, design, data acquisition and interpretation, and critically revised the manuscript. JR contributed to conception, data interpretation, and performed all statistical analyses. ZW, YZ, and PZ contributed to conception and drafted manuscript. JZ, WZ, XW, and PG contributed to data cleaning and critically revised the manuscript. All authors gave their final approval and agree to be responsible for all aspects of the work.

## Funding

This work was supported by the National Natural Science Foundation of China (Grant Nos. 81973109 and 82173588), the Project Supported by Guangdong Province Universities and Colleges Pearl River Scholar Funded Scheme (Grant No. 2019), the National Key Research and Development Program of China (Grant No. 2018YFC2000400), the Construction of High-level University of Guangdong (Grant No. G621331128), and China Postdoctoral Science Foundation funded project (Grant No. 2021M691456).

## Conflict of Interest

The authors declare that the research was conducted in the absence of any commercial or financial relationships that could be construed as a potential conflict of interest.

## Publisher's Note

All claims expressed in this article are solely those of the authors and do not necessarily represent those of their affiliated organizations, or those of the publisher, the editors and the reviewers. Any product that may be evaluated in this article, or claim that may be made by its manufacturer, is not guaranteed or endorsed by the publisher.

## References

[1] World Health Organization. The Global Prevalence of Anaemia in 2011. Geneva: WHO (2015). Available online at: https://apps.who.int/iris/handle/10665/177094 (accessed May 25, 2020).

[2] Gaskell H Derry S Andrew-Moore R McQuay HJ. Prevalence of anaemia in older persons: systematic review. BMC Geriatr. (2008) 8:1. 10.1186/1471-2318-8-1 18194534 PMC2248585

[3] Stauder R Valent P Theurl I. Anemia at older age: etiologies, clinical implications, and management. Blood. (2018) 131:505–14. 10.1182/blood-2017-07-746446 29141943

[4] De la Cruz-Góngora V Manrique-Espinoza B Villalpando S Téllez-Rojo Solís MM Salinas-Rodriguez A. Short-term impact of anemia on mortality: evidence from a sample of Mexican older adults. J Aging Health. (2014) 26:750–65. 10.1177/0898264314529331 24788718

[5] Hong CH Falvey C Harris TB Simonsick EM Satterfield S Ferrucci L. Anemia and risk of dementia in older adults: findings from the Health ABC study. Neurology. (2013) 81:528–33. 10.1212/WNL.0b013e31829e701d 23902706 PMC3775683

[6] Qin T Yan M Fu Z Song Y Lu W Fu A. Association between anemia and cognitive decline among Chinese middle-aged and elderly: evidence from the China health and retirement longitudinal study. BMC Geriatr. (2019) 19:305. 10.1186/s12877-019-1308-7 31718564 PMC6849217

[7] Hirani V Naganathan V Blyth F Le Couteur DG Seibel MJ Waite LM. Low hemoglobin concentration are associated with sarcopenia, physical performance, and disability in older Australian men in cross-sectional and longitudinal analysis: the Concord Health and Ageing in Men Project. J Gerontol A Biol Sci Med Sci. (2016) 71:1667–75. 10.1093/gerona/glw055 26994391

[8] World Health Organization. Nutritional anaemias. Report of a WHO scientific group. World Health Organ Tech Rep Ser. (1968) 405:5–37. 4975372

[9] Joosten E Detroyer E Milisen K. Effect of anaemia on hand grip strength, walking speed, functionality and 1 year mortality in older hospitalized patients. BMC Geriatr. (2016) 16:153. 10.1186/s12877-016-0326-y 27543049 PMC4992295

[10] Lanier JB Park JJ Callahan RC. Anemia in older adults. Am Fam Phys. (2018) 98:437–42. 30252420

[11] Wouters HJCM van der Klauw MM de Witte T Stauder R Swinkels DW Wolffenbuttel BHR. Association of anemia with health-related quality of life and survival: a large population-based cohort study. Haematologica. (2019) 104:468–76. 10.3324/haematol.2018.195552 30309850 PMC6395328

[12] Wu C Hu H Chou Y Huang N Chou Y Li C. What constitutes normal hemoglobin concentrations in community-dwelling older adults? J Am Geriatr Soc. (2016) 64:1233–41. 10.1111/jgs.14170 27321601

[13] Kalra PR Greenlaw N Ferrari R Ford I Tardif JC Tendera M. Hemoglobin and change in hemoglobin status predict mortality, cardiovascular events, and bleeding in stable coronary artery disease. Am J Med. (2017) 130:720–30. 10.1016/j.amjmed.2017.01.002 28109968

[14] Martinsson A Andersson C Andell P Koul S Engström G Smith JG. Anemia in the general population: prevalence, clinical correlates and prognostic impact. Eur J Epidemiol. (2014) 29:489–98. 10.1007/s10654-014-9929-9 24952166

[15] Kabat GC Kim MY Verma AK Manson JE Lessin LS Kamensky V. Association of hemoglobin concentration with total and cause-specific mortality in a cohort of postmenopausal women. Am J Epidemiol. (2016) 183:911–9. 10.1093/aje/kwv332 27076671

[16] Lee G Choi S Kim K Yun JM Son JS Jeong SM. Association between changes in hemoglobin concentration and cardiovascular risks and all-cause mortality among young women. J Am Heart Assoc. (2018) 7:e008147. 10.1161/JAHA.117.008147 30369312 PMC6201397

[17] Chueh HW Jung HL Shim YJ Choi HS Han JY on the behalf of the Red Blood Cell Disorder Working Party of The Korean Society of Hematology. High anemia prevalence in Korean older adults, an advent healthcare problem: 2007-2016 KNHANES. BMC Geriatr. (2020) 20:509. 10.1186/s12877-020-01918-9 33243179 PMC7689998

[18] Zhao L Hu C Cheng J Zhang P Jiang H Chen J. Haemoglobin variability and all-cause mortality in haemodialysis patients: a systematic review and meta-analysis. Nephrology. (2019) 24:1265–72. 10.1111/nep.13560 30644618 PMC6899589

[19] Lv Y Gao X Yin Z Chen HS Luo JS Brasher MS. Revisiting the association of blood pressure with mortality in oldest old people in China: community based, longitudinal prospective study. BMJ. (2018) 361:k2158. 10.1136/bmj.k2158 29871897 PMC5987177

[20] Zeng Y Feng Q Hesketh T Christensen K Vaupel JW. Survival, disabilities in activities of daily living, and physical and cognitive functioning among the oldest-old in China: a cohort study. Lancet. (2017) 389:1619–29. 10.1016/S0140-6736(17)30548-2 28285816 PMC5406246

[21] Shi X Yin Z Qian H Zhai Y Liu Y Xu J. A study on chronic diseases and other related health indicators of centenarians in longevity areas in China. Chin J Prev Med. (2010) 44:101–7. 10.3760/cma.j.issn.0253-9624.2010.02.004 20388328

[22] Ahmed OM Ahmed RG El-Gareib AW El-Bakry AM Abd El-Tawab SM. Effects of experimentally induced maternal hypothyroidism and hyperthyroidism on the development of rat offspring: II-the developmental pattern of neurons in relation to oxidative stress and antioxidant defense system. Int J Dev Neurosci. (2012) 30:517–37. 10.1016/j.ijdevneu.2012.04.005 22664656

[23] Little RJA Rubin DB. Statistical analysis with missing data. J Market Res. (1989) 26:374–5. 10.2307/3172915

[24] Zeng Y Chen H Shi X Yin Z Yang Z Gu J. Health consequences of familial longevity influence among the Chinese elderly. J Gerontol. (2013) 68:473–82. 10.1093/gerona/gls203 23064818 PMC3593617

[25] Denny SD Kuchibhatla MN Cohen HJ. Impact of anemia on mortality, cognition, and function in community-dwelling elderly. Am J Med. (2006) 119:327–34. 10.1016/j.amjmed.2005.08.027 16564775

[26] Zakai NA French B Arnold AM Newman AB Fried LF Robbins J. Hemoglobin decline, function, and mortality in the elderly: the cardiovascular health study. Am J Hematol. (2013) 88:5–9. 10.1002/ajh.23336 23044913 PMC3860593

[27] Lee G Choi S Kim K Yun JM Son JS Jeong SM. Association of hemoglobin concentration and its change with cardiovascular and all-cause mortality. J Am Heart Assoc. (2018) 7:e007723. 10.1161/JAHA.117.007723 29378732 PMC5850255

[28] Avesani CM Trolonge S Deleaval P Baria F Mafra D Faxén-Irving G. Physical activity and energy expenditure in haemodialysis patients: an international survey. Nephrol Dial Transplant. (2012) 27:2430–4. 10.1093/ndt/gfr692 22172727

[29] Kuhn V Diederich L Keller TSIV Kramer CM Lückstädt W Panknin C. Red blood cell function and dysfunction: redox regulation, nitric oxide metabolism, anemia. Antioxid Redox Signal. (2017) 26:718–42. 10.1089/ars.2016.6954 27889956 PMC5421513

[30] Bal Z Demirci BG Karakose S Tutal E Erkmen Uyar M Acar NO. Factors influencing hemoglobin variability and its association with mortality in hemodialysis patients. Sci World J. (2018) 2018:8065691. 10.1155/2018/8065691 29805324 PMC5899870

[31] Ferreira R Mendes SL Soares F Gonçalves F Monteiro P Monteiro S. Is hemoglobin variation a linear predictor of mortality in acute coronary syndrome? Clin Trials Regul Sci Cardiol. (2016) 19:9–12. 10.1016/j.ctrsc.2016.05.00528186441

[32] Braz VL de Oliveira Duarte YA Corona LP. The association between anemia and some aspects of functionality in older adults. Cien Saúde Colet. (2019) 24:3257–64. 10.1590/1413-81232018249.21142017 31508746

