# Supplementary material for: Is Hemoglobin Concentration a Linear Predictor of Mortality in Older Adults From Chinese Longevity Regions?
Source: Front Public Health. 2021 Nov 29;9:787935. doi: 10.3389/fpubh.2021.787935 (PMC8666873; doi:10.3389/fpubh.2021.787935)
Supplement: Supplementary file 1 [file Data_Sheet_1.PDF]

Supplementary Table 1. Sensitivity analyses for the relationship between HB concentrations and all-cause mortality risk in the older adults form Chinese longevity regions based on different exclusion criteria

HB Concentrations, g/dL	HR (95% CI)					
	Deaths/n	Excluding the participants who died in the first 6 months	Deaths/n	Excluding the participants who died in the first year	Deaths/n	Excluding the participants who died in the second year
< 11.0	256/385	1.25 (1.00-1.58)*	224/353	1.42 (1.14-1.77)*	116/245	1.34 (1.00-1.80)*
11.0-11.9	202/334	1.22 (0.97-1.52)	175/307	1.25 (0.99-1.57)	97/229	1.20 (0.89-1.62)
12.0-12.9	160/309	0.99 (0.78-1.24)	146/295	1.01 (0.80-1.28)	99/248	1.14 (0.85-1.53)
13.0-13.9	139/286	1.00 (reference)	129/276	1.00 (reference)	79/226	1.00 (reference)
≥ 14.0	131/360	0.79 (0.62-1.01)	123/352	0.75 (0.58-0.96)*	85/314	0.81 (0.59-1.11)

* $P \leq 0.05$

Abbreviations: HR: Hazard Ratio, CI: Confidence Interval.

Sensitivity analyses were based on the fully adjusted model

Supplementary Table 2. HRs (95% CI) for all-cause mortality according to HB concentrations in the older adults form Chinese longevity regions

HB concentrations, g/dL	Deaths/n	Model 1[a]		Model 2[b]		Model 3[c]		Model 4[d]	
		HR (95% CI)	***P***	HR (95% CI)	***P***	HR (95% CI)	***P***	HR (95% CI)	***P***
< 11.0	312/441	1.00 (reference)	-	1.00 (reference)	-	1.00 (reference)	-	1.00 (reference)	-
11.0-11.9	228/360	0.78 (0.66-0.93)	0.005	0.89 (0.75-1.06)	0.202	0.88 (0.74-1.05)	0.147	0.90 (0.75-1.08)	0.263
12.0-12.9	173/322	0.57 (0.47-0.68)	< 0.001	0.73 (0.60-0.88)	0.001	0.68 (0.56-0.82)	< 0.001	0.73 (0.59-0.89)	0.002
13.0-13.9	150/297	0.52 (0.43-0.64)	< 0.001	0.73 (0.60-0.89)	0.002	0.66 (0.54-0.81)	< 0.001	0.71 (0.57-0.87)	0.001
≥ 14.0	136/365	0.35 (0.28-0.42)	< 0.001	0.60 (0.49-0.74)	< 0.001	0.53 (0.43-0.66)	< 0.001	0.57 (0.45-0.72)	< 0.001

[a] *No adjustment.*

[b] *Adjusted for age, sex, education time, marital status, residence, and economic status.*

[c] *Additionally adjusted for smoking status, drinking status, tea drinking, food diversity score, regular exercise, restricted ADL, self-reported diagnosed hypertension, diabetes, stroke, heart disease, MMSE score , depressive symptom score.*

[d] *Additionally adjusted for BMI, CRP, eGFR, WBC count, MCV, platelet count, and TC.*

Abbreviations: HB: Hemoglobin, ADL: Activities of Daily Living, MMSE: Mini-Mental State Examination, BMI: Body Mass Index, CRP: C-Reactive Protein, eGFR: Estimated Glomerular Filtration Rate, WBC: White Blood Cell, MCV: Mean Corpuscular Volume, TC: Total Cholesterol.
